# Effects of Electronic Irradiation on the Characteristics of the Silicon Magnetic Sensitive Transistor

**DOI:** 10.3390/mi14020430

**Published:** 2023-02-11

**Authors:** Zhipeng Yu, Xiaofeng Zhao, Weiwei Liu, Susu Li, Zijiang Yang, Dianzhong Wen, Hongquan Zhang

**Affiliations:** 1 School of Electronic Engineering, Heilongjiang University, Harbin 150080, China; 2 Heilongjiang Provincial Key Laboratory of Micro-Nano Sensitive Devices and Systems, Heilongjiang University, Harbin 150080, China

**Keywords:** electron irradiation, silicon magnetic sensitive transistor, voltage magnetic sensitivity, irradiation damage

## Abstract

This work researched the effects of irradiation on the current-voltage characteristics and voltage magnetic sensitivity of the silicon magnetic sensitive transistor (SMST). The 1-MeV electron irradiation source was used to irradiate the SMST. The irradiation fluences were 1 × 10^12^ e/cm^2^, 1 × 10^13^ e/cm^2^ and 1 × 10^14^ e/cm^2^, respectively (the irradiation flux was 1 × 10^10^ cm^−2^·s^−1^). The experimental results demonstrate that the collector current (*I*_C_) of the SMST occurs attenuation after irradiation under the same collector voltage (*V*_CE_) and the base current (*I*_B_). The attenuated rate of the *I*_C_ increases obviously with the enhance of electron irradiation fluence when the *I*_B_ is the same. Moreover, the attenuated rate of the *I*_C_ increases slight with the rise of the *I*_B_ when the electron irradiation fluence is the same. When the supply voltage is 5.0 V (*R*_L_ = 1.5 kΩ) and the *I*_B_ is 4.0 mA, the voltage magnetic sensitivity (*S*_V_) of the SMST occurs attenuate after irradiation. The attenuated rate of the *S*_V_ increases with the enhance of electron irradiation fluence.

## 1. Introduction

Magnetic field measurements are crucial to explore the terrestrial and planetary magnetospheres, and to derive essential information about the surroundings of objective bodies in space [1,2]. Magnetic sensors, as the magnetic field measuring instrument, have been widely researched in order to meet applications in the space, including fluxgate magnetometer [3], spin-dependent tunnelling magnetometer [4], anisotropic magneto resistive sensors [5], giant magnetoresistance (GMR) sensors [6], Hall magnetic field sensors [7,8,9], etc. However, the space is full of a variety of high-energy charged particles and cosmic rays, which may lead to the performance degradation or failure of electronic devices, or even the breakdown of electronic equipment systems [10,11,12,13]. In earlier reports, Sanz et al. researched the effect of gamma ray irradiation on characteristics of anisotropic magneto-resistive magnetic sensors, in which the sensors with an integrated front-end can be vulnerable to radiation [14]. Abderrahmane et al. reported the effect of proton irradiation on characteristics of AlGaN/GaN micro-Hall sensors, which the result shows the degradation of the current-voltage characteristics and high resistivity after irradiation [15]. Therefore, the effect of irradiation on those magnetic sensor characteristics is not negligible in space applications.

In previous work, we used silicon magnetic sensitive transistors (SMST) and the Hall element to construct a monolithic-integrated three-dimensional magnetic field sensor chip which contributed to measurements of a space magnetic field vector [16]. The SMST, as the most important component of the chip, has a bipolar junction transistor structure. However, the bipolar transistor is prone to suffer from ionizing radiation damage and displacement radiation damage under space particle irradiation [17,18]. Liu et al. reported the effect of 20 MeV Br radiation on characteristics of the bipolar transistor, in which the current gain decreases with an increase in ion fluence [19]. Yue et al. reported the effect of the 170 KeV proton irradiation on characteristics of the bipolar transistor, which the result shows the decrease of gain and the increase of low-frequency noise in the irradiated sample [20]. For expanding the application of the chip to space magnetic field measurement, it is necessary to explore the radiation effects on its component characteristics.

The effect of Earth’s radiation belts on satellite is mainly deduced by protons and high-energy electrons, with energies of approximately 1 MeV [21]. In the laboratory, 1 MeV electron irradiation has been frequently used to research damage in semiconductors [22]. In this work, the 1-MeV electron irradiation source was used to irradiate the chip; furthermore, we researched the effects of irradiation on the *I*_C_-*V*_CE_ characteristics and voltage magnetic sensitivity of the SMST. The irradiation fluences were 1 × 10^12^ e/cm^2^, 1 × 10^13^ e/cm^2^ and 1 × 10^14^ e/cm^2^, respectively (the irradiation flux was 1 × 10^10^ cm^−2^·s^−1^). Whereafter, we discussed the damage mechanism of the *I*_C_-*V*_CE_ characteristics and voltage magnetic sensitivity. This lays the foundation for further research into the radiation hardening measures of the SMST.

## 2. Device and Experiment

### 2.1. Basic Structure

**Figure 1:**
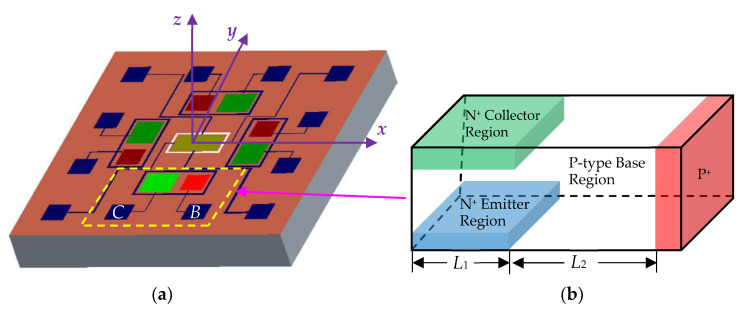
The basic structure diagrams of the device. (**a**) The magnetic field sensor; (**b**) equivalent structure diagram of SMST.

Figure 1a demonstrates the basic structure of the magnetic field sensor chip with our development previously [16]. The chip was composed of SMSTs and Hall element. Figure 1b demonstrates equivalent structure diagram of the SMST. The SMST was fabricated on p-type high resistance silicon on the basis of MEMS technology, including collector region, base region and emitter region. The n-type collector region was fabricated on the upper surface of the chip, and the silicon cup structure was etched below the collector region on the bottom surface of the chip. The n-type emitter region was fabricated in the silicon cup. The base region is p-type. The collector electrode (C) and base electrode (B) were manufactured on the upper surface of the chip, and the emitter electrode (E) was manufactured on the bottom surface of the chip. The base region of the SMST includes two parts, namely, the transport base region (the region with length *L*_1_ from Figure 1b) and the recombination base region (the region with length *L*_2_ from Figure 1b). A part of the electrons injected from the emitter junction are collected by the collector region through the transport base region to form the collector current (*I*_C_). Another part of the electrons injected from the emitter junction are recombined with the holes injected from the base in the recombination base region. SMST have a long base structure compared with general transistors, meaning the width of the base region is greater than the carrier effective diffusion length.

### 2.2. Working Principle of the SMST

**Figure 2:**
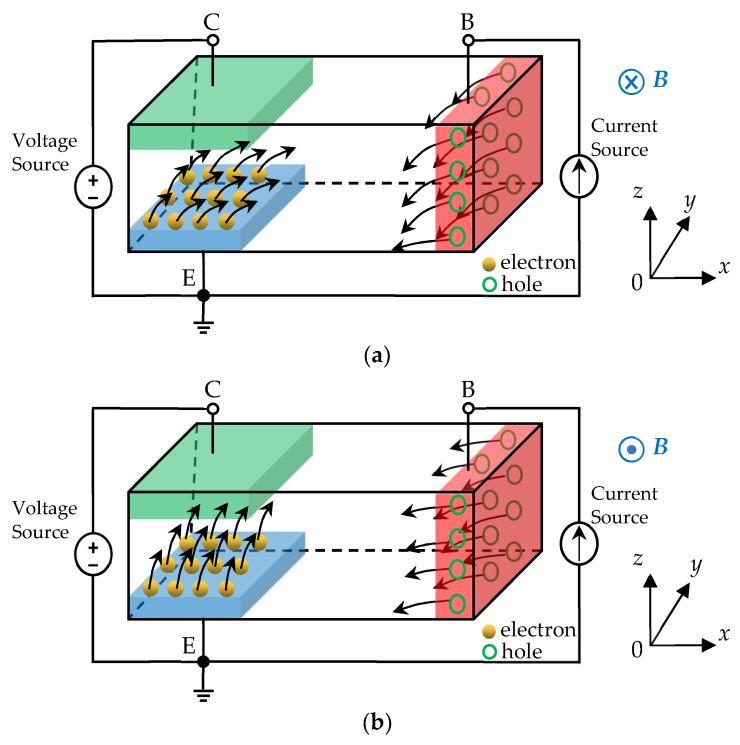
Schematic diagrams of working principle of the SMST. (**a**) *B* > 0 T; (**b**) *B* < 0 T.

Figure 2 demonstrates the working schematic diagram of the SMST [23,24]. In the space rectangular coordinate system, we placed the magnetic sensitive direction of the SMST along the *y* axis. We defined the external magnetic field along the +*y* axis as the positive magnetic field, while the external magnetic field along the −*y* axis was defined as the negative magnetic field. As shown in Figure 2a, when a positive magnetic field is applied, electrons injected into the base region from the emitter junction are deflected toward the recombination base region due to the Lorentz force, where the electrons are recombined with the holes injected from the base. Due to the modulation of the recombination base region, effective life and the effective diffusion length of the electrons are shortened. The number of electrons collected by the collector decline, resulting in the reduction of *I*_C_. As shown in Figure 2b, when a negative magnetic field is applied, electrons injected into the base region from the emitter junction are deflected toward the opposite side of the recombination base region due to the Lorentz force. Due to the weak modulation of the recombination base region to the electrons, the effective life and the effective diffusion length of the electrons extended corresponding. The number of electrons collected by the collector region rise, resulting in the increase in *I*_C_. It can be seen that *I*_C_ can be modulated by the external magnetic field, so the SMST has positive and negative magnetic sensitivity.

### 2.3. Experiment Method

We selected three samples that our laboratory had fabricated previously to conduct experiment [16], of which the sample numbers are 1#, 2# and 3#. The irradiation experiments are carried out on the high frequency and high voltage electron accelerator (DD1.2, Shanghai Xianfeng Electric machinery factory, Shanghai, China). The irradiation direction was perpendicular to the upper surface of the device (along the z-direction in Figure 1a). The irradiation fluences of sample 1#, 2# and 3# were 1 × 10^12^ e/cm^2^, 1 × 10^13^ e/cm^2^ and 1 × 10^14^ e/cm^2^, respectively. The energy of the electron irradiation source was 1-MeV, and the irradiation flux was 1 × 10^10^ cm^−2^·s^−1^. The test equipment were semiconductor characterization system (Keithley 4200, Tektronix, Beaverton, OR, USA), data multimeter (34461A, Keysight, Santa Rosa, CA, USA), constant voltage source (BJ1790B, Beijing Radio Instrument Factory, Beijing, China), constant current source (DP832, RIGOL, Beijing, China) and magnetic field generator system (CH-100, CH-Magnetoelectricity Technology, Beijing, China). The parameters of irradiated sample could be tested within 30 min after irradiation. 

## 3. Results and Discussion

### 3.1. I_C_-V_CE_ Characteristics of the SMST

**Figure 3:**
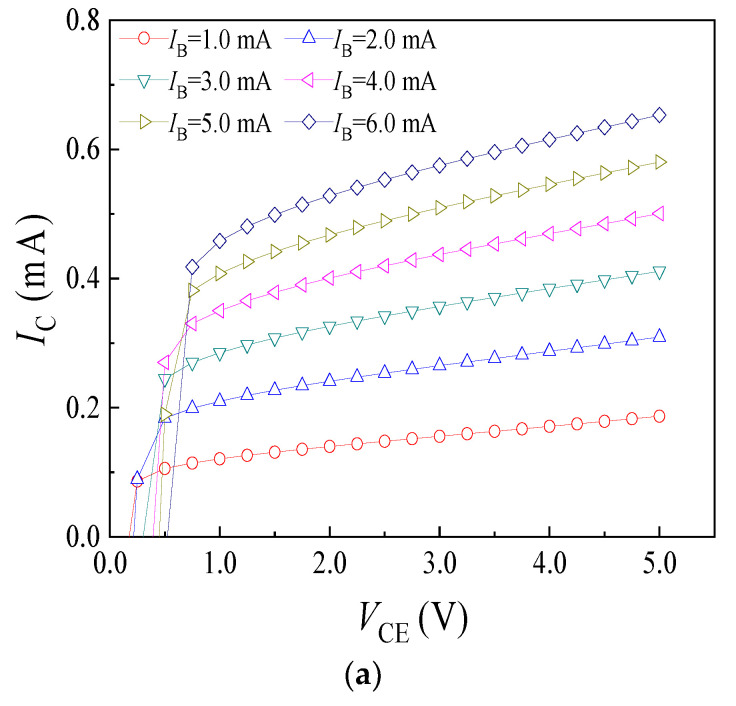

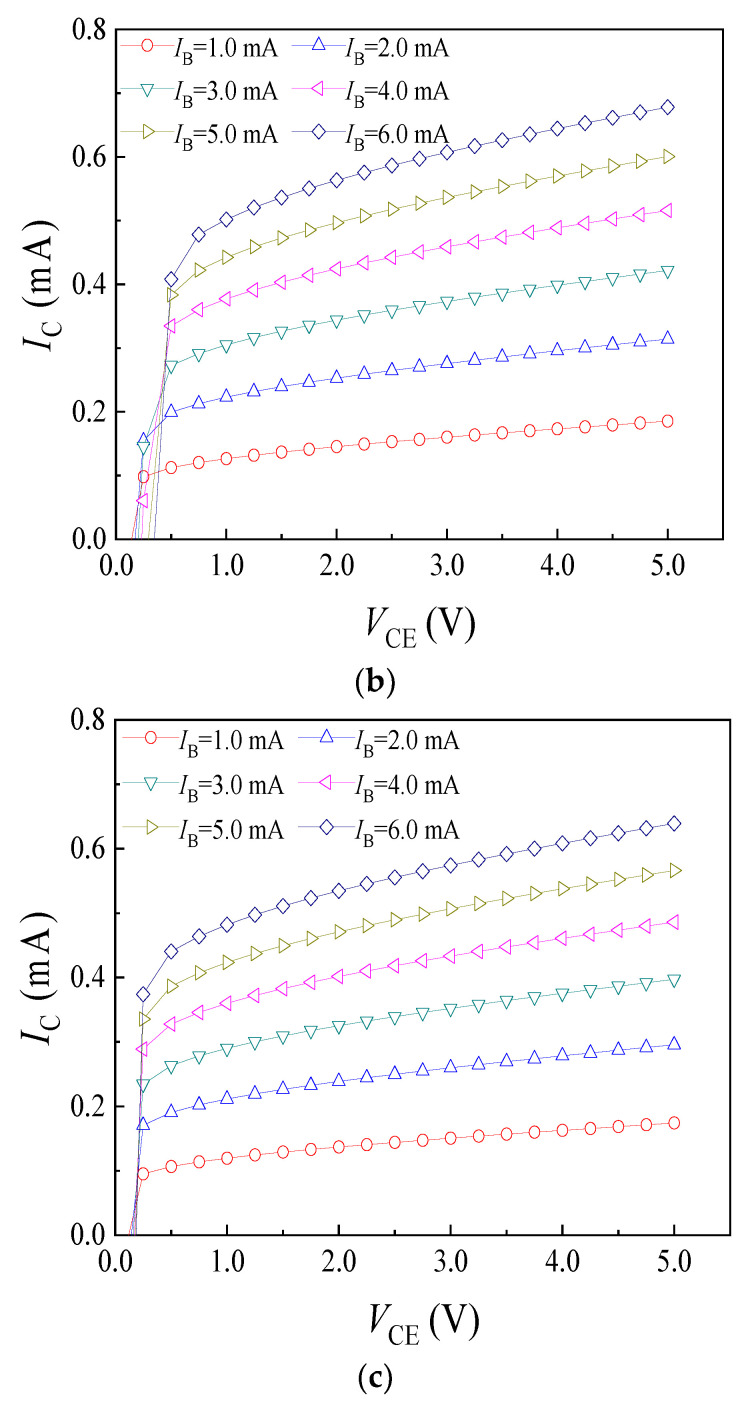
*I*_C_-*V*_CE_ characteristic curves of the SMSTs. (**a**) Sample 1#; (**b**) sample 2#; (**c**) sample 3#.

At room temperature, the *I*_C_-*V*_CE_ characteristics of three samples were tested before electron irradiation. Figure 3 demonstrates the *I*_C_-*V*_CE_ characteristic curves of three samples without an external magnetic field. As shown in Figure 3a, the curves rise inclined when the *I*_B_ is constant. The *I*_C_ increases significantly with the rise of the *V*_CE_, because the ability of that collector region collect electrons is enhanced. As the *V*_CE_ further increases, the ability of that collector region collect electrons reaches saturation. Ideally, the *I*_C_ should remain constant. However, due to the base width modulation effect [25], the rise of *V*_CE_ will lead to the expansion of the depletion layer of the collector junction towards both sides of the base region and the collector region, reducing the base region width. As a result, the carrier recombination rate in the base region declines and the electron concentration gradient increases, resulting in a small increase in *I*_C_ with the rise of *V*_CE_. When the *V*_CE_ is constant, the *I*_C_ increases with the rise of *I*_B_, but the increment of *I*_C_ will gradually decrease with the rise of the *I*_B_, which could be caused by the large injected-charge density [26]. The above analysis shows that the *I*_C_-*V*_CE_ characteristics of the SMST is similar to general transistors, but the value of *I*_C_ is always less than the *I*_B_, due to the wide base structure. Through comparative analysis, sample 2# and 3# have similar *I*_C_-*V*_CE_ characteristic with sample 1#, as shown in Figure 3b,c.

### 3.2. Effect of Electron Irradiation on I_C_-V_CE_ Characteristics of the SMST

**Figure 4:**
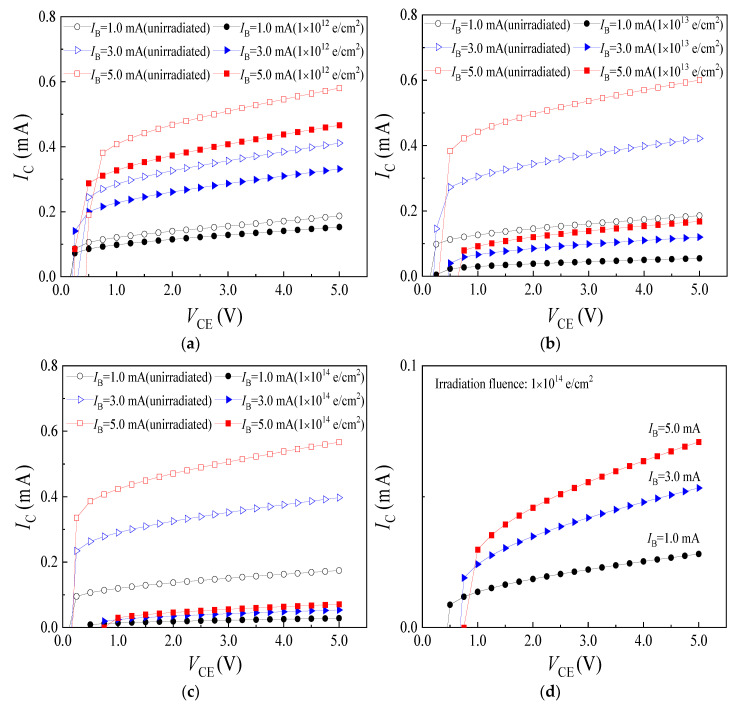
*I*_C_-*V*_CE_ characteristic curves of the SMSTs before and after electron irradiation. (**a**) Sample 1#; (**b**) sample 2#; (**c**) sample 3#; (**d**) highlight for irradiated sample 3#.

The *I*_C_-*V*_CE_ characteristic curves of sample 1#, 2# and 3# before and after electron irradiation were shown in Figure 4a–c, respectively. In addition, Figure 4d highlights the *I*_C_-*V*_CE_ characteristic curves of irradiated sample 3#. After the SMST were irradiated by electrons, the *I*_C_ corresponding to the same *V*_CE_ and *I*_B_ in the curve saturation region have attenuation. When the irradiation fluences were 1 × 10^12^ e/cm^2^ and 1 × 10^13^ e/cm^2^, the *I*_C_ attenuated obviously, but the *I*_C_-*V*_CE_ characteristic curves remain in normal shape without failure. After the SMST was irradiated by electrons with irradiation fluence of 1 × 10^14^ e/cm^2^, the value of *I*_C_ was very small. At this moment, the SMST tends to fail.

In order to further research the effects of irradiation fluence and *I*_B_ on the damage degree of *I*_C_ after electron irradiation, the *I*_C_ attenuation rate of the SMST were calculated when *V*_CE_ = 5.0 V. The calculation formula is shown as the following:

(1) $$D_{\mathrm{IC}}=\frac{\left|I_{\mathrm{Cpost}}-I_{\mathrm{Cpre}}\right|}{I_{\mathrm{Cpre}}}\times 100\%$$

where *D*_IC_ is the attenuation rate of *I*_C_, *I*_Cpost_ is the collector current of the SMST after irradiation and *I*_Cpre_ is the collector current of the unirradiated SMST.

The calculation results of attenuation rate of *I*_C_ were given in Table 1. The attenuation rate of *I*_C_ increases slight with the rise of *I*_B_ under the same irradiation fluence. At the same *I*_B_, the attenuation rate of *I*_C_ increases obviously with the enhance in irradiation fluence. In summation, the damage degree of *I*_C_ increases gradually with the enhance of electron irradiation fluence and *I*_B_ after irradiation.

### 3.3. Effect of Electron Irradiation on Voltage Magnetic Sensitivity of the SMST

**Figure 5:**
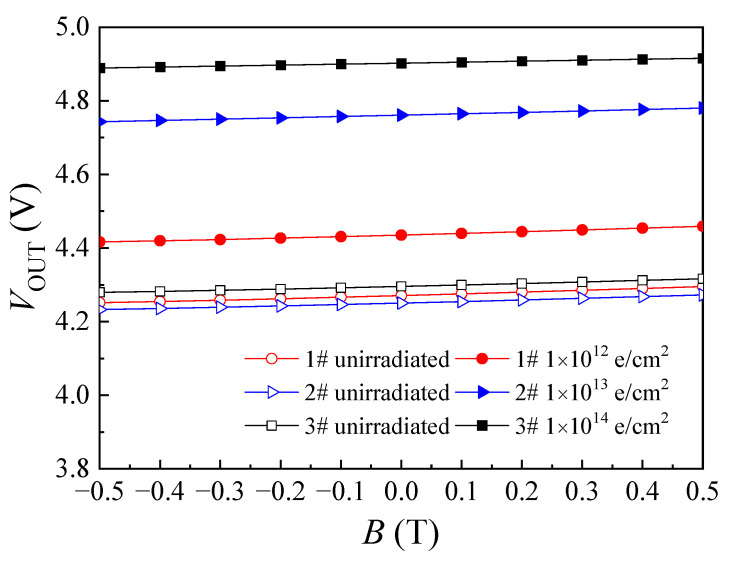
Relation curves of *V*_OUT_ and *B* of SMSTs before and after electron irradiation.

To analyze voltage magnetic sensitivity, we build the test circuit which the collector electrode of the SMST was connected the load resistance *R*_L_ (the value of resistance is 1.5 kΩ) and the voltage source (*V*_DD_ = 5.0 V) was applied on the other side of the *R*_L_; the current source (*I*_B_ = 4.0 mA) was connected between the base electrode and the emitter electrode when the emitter electrode was grounded. Figure 5 demonstrates the relation curves of the collector output voltage (*V*_OUT_) for SMSTs and the applied magnetic field (*B*) before and after electron irradiation.

According to magnetic sensitive characteristic of the SMST, the value of *I*_C_ can be modulated by applied magnetic field, so the *V*_OUT_ will change corresponding. In Figure 5, it can be seen that the value of *V*_OUT_ for the irradiated three samples increase obviously under the same applied magnetic field, and the increment of *V*_OUT_ enlarges with the increase in the electron irradiation fluence. On the basis of the definition of the sensor sensitivity, the voltage magnetic sensitivity was calculated using the following formula [23]:

(2) $$S_{V}=\frac{\left|V_{\mathrm{OUT}+}-V_{\mathrm{OUT}-}\right|}{B}$$

where *S*_V_ is the voltage magnetic sensitivity, *V*_OUT+_ is the collector output voltage under applied positive magnetic field, *V*_OUT-_ is the collector output voltage under applied negative magnetic field and *B* is the applied magnetic field.

To further research the effect of electron irradiation on the voltage magnetic sensitivity of the SMST, the attenuation rate of the voltage magnetic sensitivity was calculated using the following formula:

(3) $$D_{\mathrm{SV}}=\frac{\left|S_{\mathrm{Vpost}}-S_{\mathrm{Vpre}}\right|}{S_{\mathrm{Vpre}}}\times 100\%$$

where *D*_SV_ is the attenuation rate of the voltage magnetic sensitivity, *S*_Vpost_ is voltage magnetic sensitivity of sample after irradiation, *S*_Vpre_ is the voltage magnetic sensitivity of unirradiated sample.

Table 2 shows the calculation results of the attenuation rate of the voltage magnetic sensitivity. The voltage magnetic sensitivity of the SMST occurred attenuation after irradiation, and the attenuation rate of the voltage magnetic sensitivity increases with the enhance in the irradiation fluence. 

### 3.4. Discussion on Damage Mechanism

When energetic particles irradiate semiconductor materials, they lose their energy by ionizing and nonionizing processes; the results point to ionization damage (generating electron-hole pairs) and displacement damage (generating Frenkel pair, divacancy and defect complexes) [27]. Ionization damage mainly influences the insulating layer and Si/SiO_2_ interface, while displacement damage primarily influences silicon substrate in bipolar transistors [28]. The SMST as a kind of minority carrier device, in which the carrier transport occurs mainly in silicon bulk, is susceptible to displacement damage [19]. After irradiation, a variety of deep and shallow defects were introduced in the silicon, on account of displacement damage, of which, shallow defects can compensate for majority carriers, resulting in the reduction of carriers, and deep defects can be used as carrier generation, recombination and capture centers, which will reduce the minority carrier lifetime [29]. In the operating conditions, electrons are injected into a p-type base region from the emission junction. In the base region, electrons are also captured by traps and recombination centers, in addition to recombination with holes injected from the base. As a result, the effective lifetime and diffusion length of electrons in the base region are reduced compared with that before irradiation. This will lead to a reduction in the number of electrons collected by the collector, resulting in a reduction in *I*_C_. Therefore, traps and recombination center may be one of main reasons for the reduction of *I*_C_. The modulation effect of the external magnetic field to the carriers mainly occurs in the base region. The traps and recombination centers introduced by radiation can reduce the effective lifetime, shortening the effective diffusion length and reducing the mobility of electron in base region [30]. These factors can reduce the response ability of carriers to the external magnetic field in the SMST, resulting in the reduction of voltage magnetic sensitivity. With the rise of irradiation fluence, the concentration of traps and recombination centers in the base region increases, resulting in more severe attenuation of *I*_C_ and voltage magnetic sensitivity.

## 4. Conclusions

In this work, we researched the effects of 1-MeV electron irradiation on the *I*_C_-*V*_CE_ characteristics and voltage magnetic sensitivity of the SMST. Compared with before irradiation, the *I*_C_ of the SMST is attenuated after irradiation. When the *I*_B_ is the same, the attenuated degree of *I*_C_ increases obviously with the enhance of electron irradiation fluence. In addition, the rise of *I*_B_ can lead to the slight increase of attenuated degree of *I*_C_ when the electron irradiation fluence is the same. In the same operating condition, the voltage magnetic sensitivity of the SMST is attenuated after irradiation. With the enhance in irradiation fluence, the attenuation degree of voltage magnetic sensitivity of the SMST increases. The above work lays a foundation for further research of hardening measures of SMST in irradiation environment application.

## Figures and Tables

**Table 1:** Attenuation rate of *I*c for SMST after irradiation.

Sample	Irradiated Fluences (e/cm^2^)	*D*_IC_ (*I*_B_ = 1.0 mA)	*D*_IC_ (*I*_B_ = 2.0 mA)	*D*_IC_ (*I*_B_ = 3.0 mA)	*D*_IC_ (*I*_B_ = 4.0 mA)	*D*_IC_ (*I*_B_ = 5.0 mA)	*D*_IC_ (*I*_B_ = 6.0 mA)
1#	1 × 10^12^	18.151%	18.970%	19.379%	19.606%	19.723%	19.724%
2#	1 × 10^13^	70.505%	71.313%	71.663%	71.879%	72.032%	72.158%
3#	1 × 10^14^	83.869%	85.712%	86.572%	87.103%	87.481%	87.770%

**Table 2:** Attenuation rate of voltage magnetic sensitivity for SMST after irradiation.

Sample	Irradiated Fluences (e/cm^2^)	*D*_SV_
1#	1 × 10^12^	2.1%
2#	1 × 10^13^	6.8%
3#	1 × 10^14^	28.8%

## Data Availability

Not applicable.
